# Genome-wide atlas of alternative polyadenylation in the forage legume red clover

**DOI:** 10.1038/s41598-018-29699-7

**Published:** 2018-07-27

**Authors:** Manohar Chakrabarti, Randy D. Dinkins, Arthur G. Hunt

**Affiliations:** 1 Department of Plant and Soil Sciences, University of Kentucky, Lexington, Kentucky 40546-0312 USA; 2 USDA-ARS, Forage-Animal Production Research Unit, Lexington, Kentucky 40546 USA

## Abstract

Studies on prevalence and significance of alternative polyadenylation (APA) in plants have been so far limited mostly to the model plants. Here, a genome-wide analysis of APA was carried out in different tissue types in the non-model forage legume red clover (*Trifolium pratense* L). A profile of poly(A) sites in different tissue types was generated using so-called ‘poly(A)-tag sequencing’ (PATseq) approach. Our analysis revealed tissue-wise dynamics of usage of poly(A) sites located at different genomic locations. We also identified poly(A) sites and underlying genes displaying APA in different tissues. Functional categories enriched in groups of genes manifesting APA between tissue types were determined. Analysis of spatial expression of genes encoding different poly(A) factors showed significant differential expression of genes encoding orthologs of FIP1(V) and PCFS4, suggesting that these two factors may play a role in regulating spatial APA in red clover. Our analysis also revealed a high degree of conservation in diverse plant species of APA events in mRNAs encoding two key polyadenylation factors, CPSF30 and FIP1(V). Together with our previously reported study of spatial gene expression in red clover, this study will provide a comprehensive account of transcriptome dynamics in this non-model forage legume.

## Introduction

Polyadenylation is a post-transcriptional processing step essential for the maturation of the majority of the eukaryotic messenger RNAs (mRNAs). Except for replication-dependent transcripts encoding histone genes in metazoans, all precursor-mRNAs (pre-mRNAs) undergo polyadenylation^1,2^. Along with mRNAs, other RNA polymerase II-encoded transcripts, such as long non-coding RNAs (lncRNAs) also bear poly(A) tails^3^. Polyadenylation process typically involves recognition of the poly(A) signal present in the pre-mRNA by a multi-protein complex, endo-nucleolytic cleavage of the nascent mRNAs at the cleavage and polyadenylation site [poly(A) site, PAS] and subsequent addition of un-templated poly(A) tails to the 3′end of the cleaved transcripts by the poly(A) polymerase (PAP)^2,3^. Poly(A) tails at the 3′ ends of mature mRNAs regulate their stability, export to cytosol, and translation^4,5^.

A gene may contain multiple poly(A) sites (PASs) and differential usage of these PASs gives rise to distinct transcripts. This phenomenon is termed as ‘alternative polyadenylation’ (APA)^2,3^. Along with alternative transcription initiation and alternative splicing, APA can contribute to enlarging the transcriptome and proteome complexity. Earlier genome-wide studies using expressed sequence tags (ESTs) and recent high-throughput sequencing studies revealed the widespread prevalence of APA in diverse organisms, including mammals, yeast and nematodes^6–11^. High-throughput studies have also revealed genome-wide occurrences of APA in several plant species, including Arabidopsis, rice, and Medicago^12–16^.

There are significant implications of APA in numerous biological processes. Mis-regulation of APA has been attributed as the cause for several human diseases, including α-thalassemia, β-thalassemia, thrombophilia, metachromatic leukodystrophy, IPEX syndrome, oculopharyngeal muscular dystrophy, and preeclampsia^17–26^. A global increase in the usage of proximal PAS was observed in case of cardiac hypertrophy and in cancer cells^27,28^. In plants, APA has been associated with the regulation of flowering time, self-incompatibility, seed dormancy, amino acid catabolism, and legume-rhizobia symbiosis^29–37^. APA may also occur in a tissue-specific fashion to regulate tissue-specific developmental processes. Tissue-specific occurrences of APA have been demonstrated in human, *Drosophila*, and *Caenorhabditis elegans*^9,11,38–40^. For instance, in human mRNA isoforms in blood, and testis tend to be derived proximal PASs, whereas mRNA isoforms in neuronal tissues predominantly are derived from use of distal PASs. This pattern of preferential usage of proximal or distal PASs by the mRNA isoforms in testis, and brain is also found to be conserved in *Drosophila*^8,38,39,41^. In plants, occurrences of tissue-specific APA so far have been demonstrated only in model plants *Arabidopsis* and rice^12,15,42^. Here, we report the genome-wide tissue-specific atlas of APA in red clover (*Trifolium pratense* L.), a cool season forage legume. Red clover is the second most widely grown forage legume in the United States, after alfalfa, and is considered a high-value feed for livestock because of its digestibility, and protein content^43^. Being a nitrogen-fixing forage crop, red clover has a great potential in sustainable agriculture^44,45^. However, genomic resources for red clover are scarce, save for a genome sequence and a few transcriptome studies^46–48^. Together with the spatial gene expression analysis, the tissue-wide global APA analysis provides a comprehensive understanding of transcriptional dynamics, and of the role of APA in fine-tuning transcriptome plasticity in this important forage legume.

## Results

### PATseq library preparation, next generation sequencing, profiling of poly(A) sites and their validation

To decipher the spatial dynamics of genome-wide alternative polyadenylation in red clover, three different tissue types were studied. PAT libraries were sequenced using Illumina platform and after demultiplexing individual libraries, altogether 53.6 million reads were obtained. Next-generation sequencing data generated in this project was submitted to the NCBI Short Read Archive (http://www.ncbi.nlm.nih.gov/sra) under the BioProject accession PRJNA412508. After trimming Illumina adapter sequences and poly(T) tract at the start of each PAT reads, roughly 49.5 million reads were retained. Trimmed PATs were mapped to the red clover genome, which resulted in approximately 17.5 million mapped reads with an average of 1.9 million mapped reads per library. A summary of mapped reads for each library is presented in the Supplemental Table S1.

For the genome-wide APA analysis, sequencing reads were trimmed to one nt tags and processed as described in Methods, yielding sets of poly(A) sites (PASs) and poly(A) site clusters (PACs). Slightly more than 93,000 PASs and approximately 28,000 PACs were defined by the mapped PATs; these are listed in Supplemental Dataset S1 and S2, respectively. The collection of PACs maps to 12,413 different annotated genes.

To further validate the PACs generated using the PATseq dataset, an independent RNAseq dataset [NCBI-SRA BioProject accession PRJNA287846] was used. Poly(A)-containing reads were extracted from the RNAseq dataset and these reads were used to generate a PAC list. PACs lists generated using the PATseq and RNAseq datasets were compared to assess overlap between the genomic coordinates of PACs in two datasets. As expected very few poly(A) tail-containing reads were extracted from the RNAseq dataset (Supplemental Table S2). However, using these reads, 241 PACs could be defined. Out of these 176 PACs overlap with the PACs generated using the PATseq approach (Supplemental Dataset S3). This analysis confirms that most of the PACs identified with PATs are valid.

As a further quality control step, gene-by-gene comparisons of poly(A) tag distributions were performed using a previously described tool, PATAPP^49^. PATAPP calculates fractional usage of individual PASs for each library, and subsequently, compares these values between two contrasting libraries on a gene-by-gene basis. A poly(A) metric of ‘0’ and ‘1’ define absolute similarity and dissimilarity, respectively. The PATAPP analysis also revealed close correspondence between different replicates of each tissue samples (Supplemental Fig. S1).

To further assess the reproducibility of the individual sequencing samples generated in this study, gene expression levels were estimated by determining the numbers of individual PATs that map to annotated genes, and the results were assessed using a correlation scatterplot (Supplemental Fig. S2). This plot showed a good correspondence between individual replicates from the three tissues, and revealed that the leaf and flower tissues were more similar to each other than they were to root tissues. Additionally, Pearson correlation coefficients between replicates of each tissue sample were found to be between 0.85–0.92, suggesting good correspondence among replicates of each tissue type (Supplemental Table S3). These results provide further demonstration of the utility of the sequencing data.

### Distribution of canonical and non-canonical poly(A) sites in red clover

**Figure 1:**
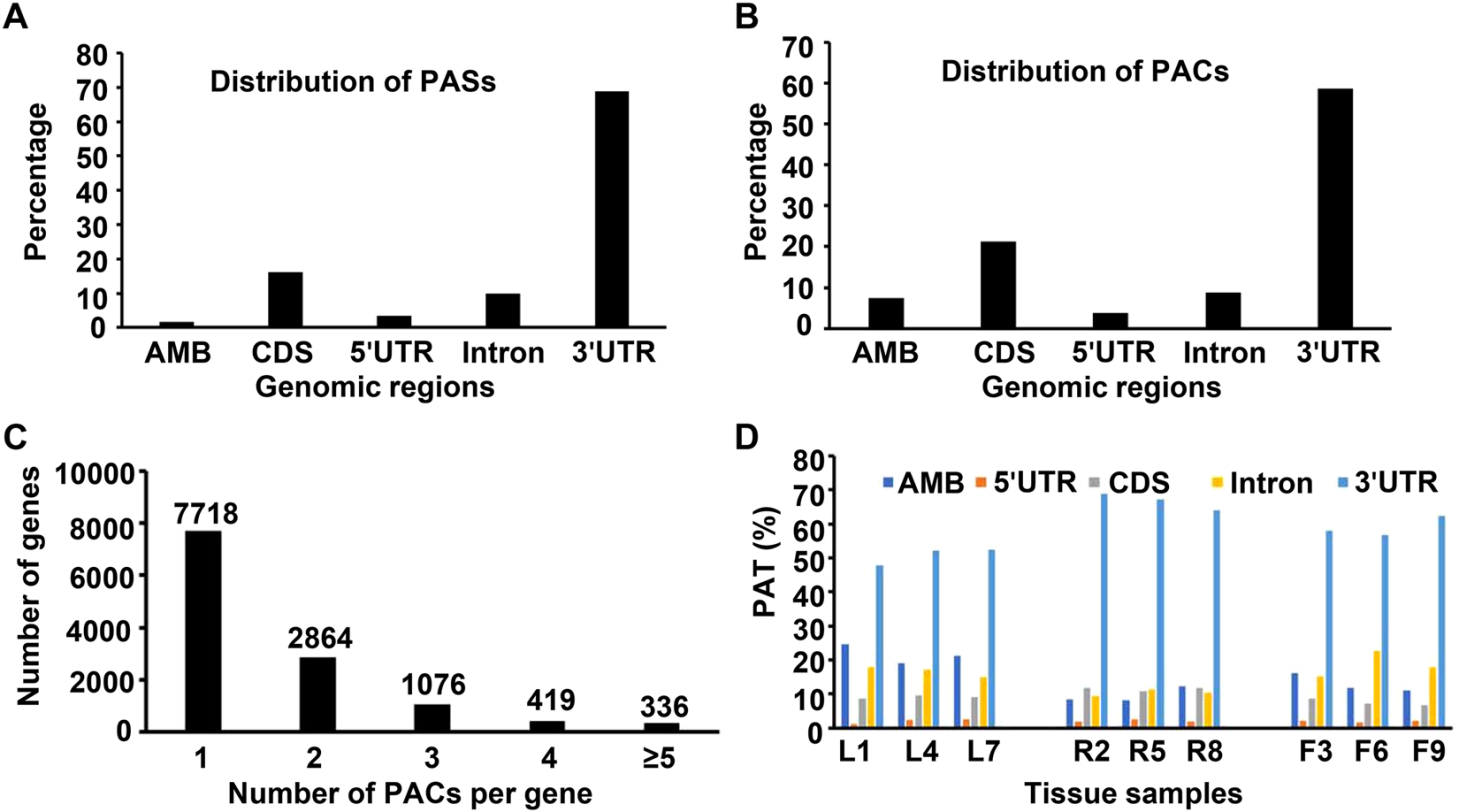
Genome-wide distribution of PASs, and PACs across different genomic regions and genomic distribution of PATs across different tissue samples. (**A**) Distribution of PASs and associated PATs across different genomic regions. (**B**) Distribution of PACs and associated PATs across different genomic regions. (**C**) Distribution of PACs per gene. Numbers above each bar represent number of genes in each category. (**D**) Changes in the tissue-wise genomic distribution of PATs. AMB, ambiguous sites, which were assigned to multiple genomic regions. L, R, and F denote leaf, root, and flower tissue, respectively. 1 to 9 represent sample IDs.

To determine the genomic distribution of PASs and PACs, those PASs and PACs that mapped to the annotated regions of the red clover genome were counted. 68.9, 16.1, 3.4, and 9.8% of all PASs mapped to the extended 3′UTRs, protein coding regions (CDSs), 5′UTRs, and introns, respectively (Fig. 1A). Similarly, most of the PACs were mapped to the 3′UTRs, constituting about 58.6% of the total PACs, whereas, 21.3, 3.9, and 8.8% of all PACs were mapped to the CDSs, 5′UTRs, and introns, respectively (Fig. 1B). 4,695 genes, representing around 11.5% of all the red clover genes, were found to contain multiple PACs (Fig. 1C).

To decipher any spatial bias in the abundance of various mRNA isoforms, such as mRNA isoforms terminating at canonical poly(A) sites located at 3′UTRs or at non-canonical poly(A) sites (residing at other genomic locations, such as CDSs, introns and 5′UTRs), the distribution of PATs across different genomic locations in different tissue types were estimated (Fig. 1D). There was no obvious difference in the distribution of PATs across different genomic locations between leaf and flower tissues. However, distributions of PATs across various genomic locations differed in root tissue as compared to the leaf and flower tissues. Specifically, the proportion of PATs that mapped to the CDSs and 3′UTRs increased, while those that mapped to the intronic regions decreased in root tissue as compared to leaf and flower tissues (Fig. 1D).

### Tissue-wise dynamics of poly(A) site usage

**Figure 2:**
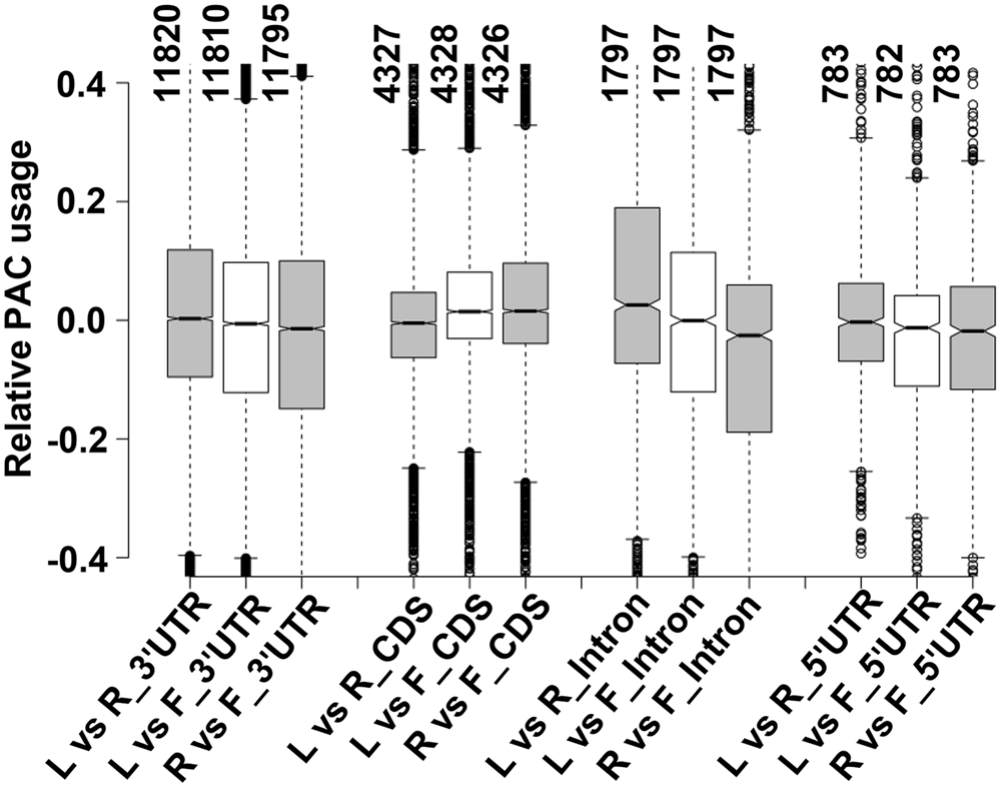
Relative PAC usage across different tissue types. Boxplots represent comparison of PAC usage between two tissue samples for different categories of transcripts, which were terminated at poly(A) sites located at various genomic regions. Y-axis represents relative PAC usage. Number of PACs used for the PAC usage analysis for 3′UTR, CDS, intron, and 5′UTR regions are represented with the vertical numbers at the top.

We have explored tissue-wise dynamics of usage of individual PACs located in different genomic locations. Poly(A) site usage was determined by the numbers of reads mapping to an individual PAC as a fraction of the total PATs that mapped to the associated gene. The difference in poly(A) site usage for each individual PAC between two tissue-types was then calculated. The aggregate differences for PACs located in different regions (3′UTRs, CDS, etc.) were then represented in boxplots (Fig. 2**)**. The most striking difference in the poly(A) site usage was observed in poly(A) sites located at introns, where poly(A) site usage exhibited a decrease in root tissue as compared to the leaf and flower tissues, while no change was observed between leaf and flower tissues (Fig. 2). This result suggests that, in genes with multiple poly(A) sites, including at least one that lies within introns, usage of the intronic sites are somewhat lower than other sites.

### Nucleotide compositions around poly(A) sites are conserved across tissue types

**Figure 3:**
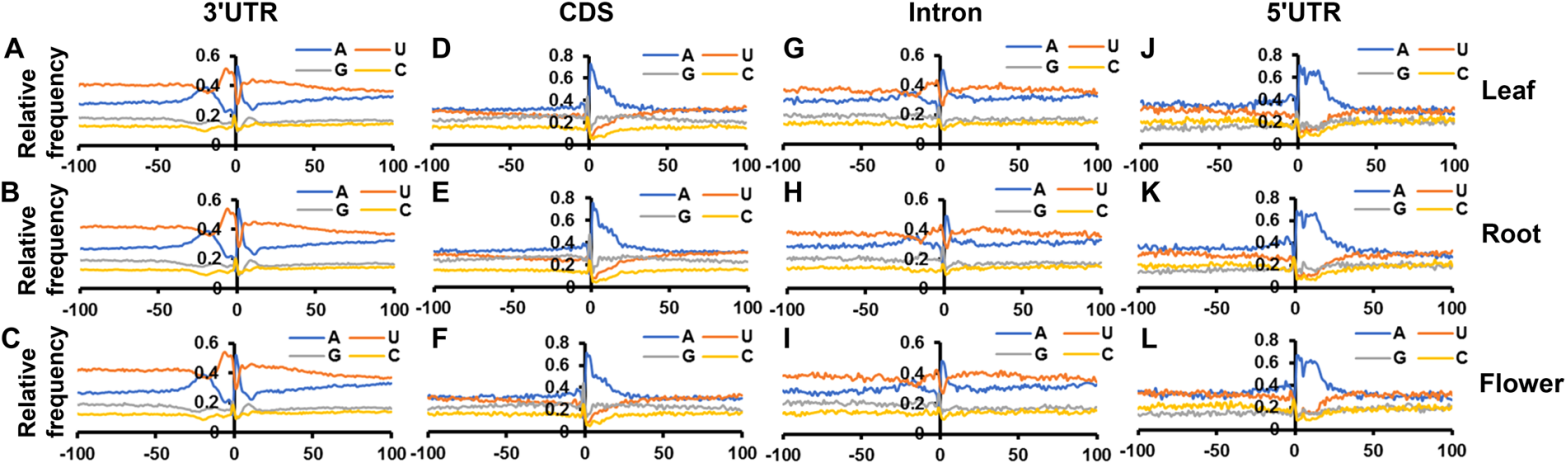
Nucleotide composition of the sequences surrounding PASs. PASs were grouped according to their genomic locations. Sequences 100 nucleotides upstream and downstream of PASs were used for the analysis. (**A**–**C**) Represent single nucleotide profiles of the sequences surrounding PASs mapped to 3′UTR regions in leaf, root, and flower, respectively. (**D–F**) Represent single nucleotide profiles of the sequences surrounding PASs mapped to CDS regions in leaf, root, and flower, respectively. (**G–I**) Represent single nucleotide profiles of the sequences surrounding PASs mapped to intronic regions in leaf, root, and flower, respectively. (**J–L**) Represent single nucleotide profiles of the sequences surrounding PASs mapped to 5′UTR regions in leaf, root, and flower, respectively. Y-axis represents relative frequency of each nucleotide.

The recognition of polyadenylation signals by polyadenylation factors is a vital step in the process of cleavage and polyadenylation of nascent transcripts. Thus, variations in polyadenylation signals may explain alternative poly(A) site usage between different samples. To assess any spatial bias in polyadenylation signals among different tissue types studied, the single nucleotide compositions of the sequences surrounding PASs utilized in different genomic regions was determined. Sequences covering 100 nucleotides upstream to 100 nucleotides downstream of PASs were used for computing single nucleotide compositions. Single nucleotide compositions of the sequences surrounding PASs located in 3′UTRs display typical patterns observed in other plant species, including U-rich upstream regions, an A-rich peak around −20 (termed as ‘NUE’), a U-rich peak immediately following the A-rich peak, and YA at the cleavage site (where, Y = U or C) (Fig. 3A–C)^12–14,50^. Single nucleotide compositions of the sequences around PASs residing at intronic regions also exhibited similar patterns observed in case of PASs located at 3′UTRs. Similar patterns were also observed in other plant species, including Arabidopsis, Medicago, and rice (Fig. 3G–I)^13,14,49^. As opposed to the PASs located at 3′UTRs and introns, PASs positioned at the protein coding regions displayed distinctly different patterns, such as presence of (A + G)-rich region in case of PASs located at protein coding regions, instead of A-rich NUE and the following U-rich peak in PASs residing at 3′UTRs and introns (Fig. 3D–F). Patterns observed in PASs located at protein coding in regions also resemble results previously reported in other plant species^12–14^.

The relatively high ‘A’ content surrounding CDS-situated PASs raises the possibility that the reads that define these sites might arise via internal priming by the reverse transcriptase. To test this possibility, the nucleotide profiles surrounding positions in the red clover genome consisting 8 or more ‘A’s (the most likely sites of internal priming) were determined, with a focus on sites located within 3′UTRs and CDS. As shown in Supplemental Fig. S3A,B, the nucleotide profiles of sequences surrounding such potential internal priming sites were completely different from the nucleotide profiles of PASs defined by PATs and located at 3′UTRs and coding regions (Fig. 3A–F). This result indicates that the sites located in CDS, in particular, are likely not derived via internal priming.

Single nucleotide compositions around PASs positioned at 5′UTRs have only been reported in rice, where it resembles the nucleotide profile observed in PASs located at 3′UTRs^14^. Similar to the profiles described in rice, we also observed A and U-rich regions upstream of PASs, low G-content as compared to the PASs residing at protein coding regions. However, we do not observe A-rich peak at the NUE and the following U-rich peak described in rice (Fig. 3J–L)^14^. This discrepancy may arise due to low number of PASs located at 5′UTRs in both studies.

### Identification of PACs displaying differential usage between tissue types

**Figure 4:**
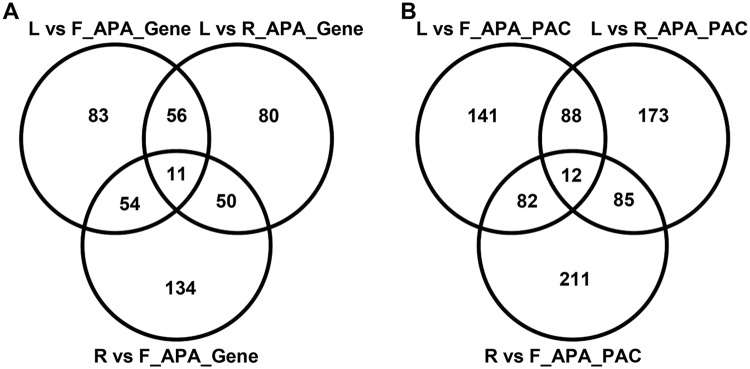
PACs and genes displaying APA between different tissue types. (**A**,**B**) Represent number of genes and PACs displaying APA between two tissue types. L, R, and F represent leaf, root, and flower, respectively. PACs and genes displaying APA between tissue types are represented in Dataset. S4 through S6.

**Figure 5:**
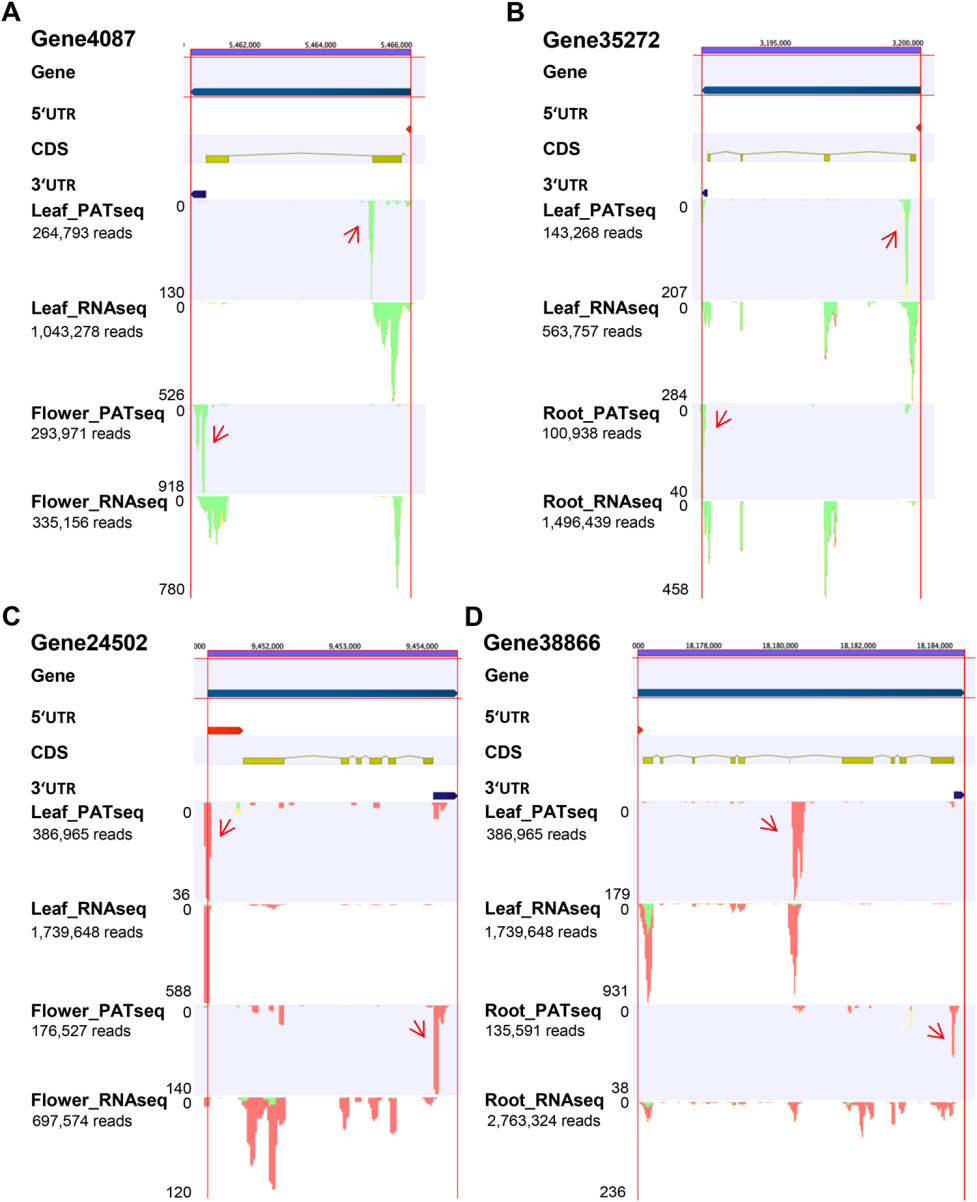
Screenshots of genes displaying APA between two tissue types. Mappings from the PATseq and RNAseq reads are represented for each gene. APA events are validated with independent RNAseq read mappings (NCBI-SRA BioProject PRJNA287846). APA events are shown with red arrows. Red vertical lines demarcate each gene.

To further delve into the spatial shifts in the poly(A) site usage, differentially utilized PACs and genes associated with such PACs were identified and characterized (Fig. 4A,B). Altogether, 792 PACs defined by 468 genes displayed tissue-wise alteration in poly(A) site usage. Among these differentially utilized PACs, 66.3% were present only in one tissue-wise comparison, whereas 33.7% PACs were present in two or three of the tissue-wise comparisons (Fig. 4B). Examples of APA events between tissue types are represented in the Fig. 5A–D. The set of genes displaying APA between tissue types included gene4087, which encodes a ‘NAD(P)-binding rossmann-fold superfamily protein’. For the gene4087, the proximal, non-canonical poly(A) site was used in the leaf tissue, whereas, in the flower tissue the distal, the canonical poly(A) site was used (Fig. 5A). The gene35272, which encodes a LEA (Late Embryogenesis Abundant) protein, displayed APA between the leaf and root tissues. For the gene35272, the proximal non-canonical poly(A) site was used in the leaf tissue, whereas, the distal, the canonical site was used in the root tissue (Fig. 5B). The gene24502, which encodes a ‘calcium-binding EF hand family protein’, exhibited APA between the leaf and flower tissues. For the gene24502, in the leaf tissue a proximal, non-canonical poly(A) site was used predominantly, whereas, in the flower tissue the distal, the canonical poly(A) site was used primarily (Fig. 5C). The gene38866, which encodes a ‘major facilitator superfamily protein’ displayed APA between the leaf and root tissues. For the gene38866, in the leaf tissue a proximal, non-canonical poly(A) site was used predominantly, whereas, in the root tissue mainly the distal, canonical poly(A) site was used (Fig. 5D). These APA events were also confirmed with a previously reported independent RNAseq dataset (Fig. 5A–D)^48^.

**Figure 6:**
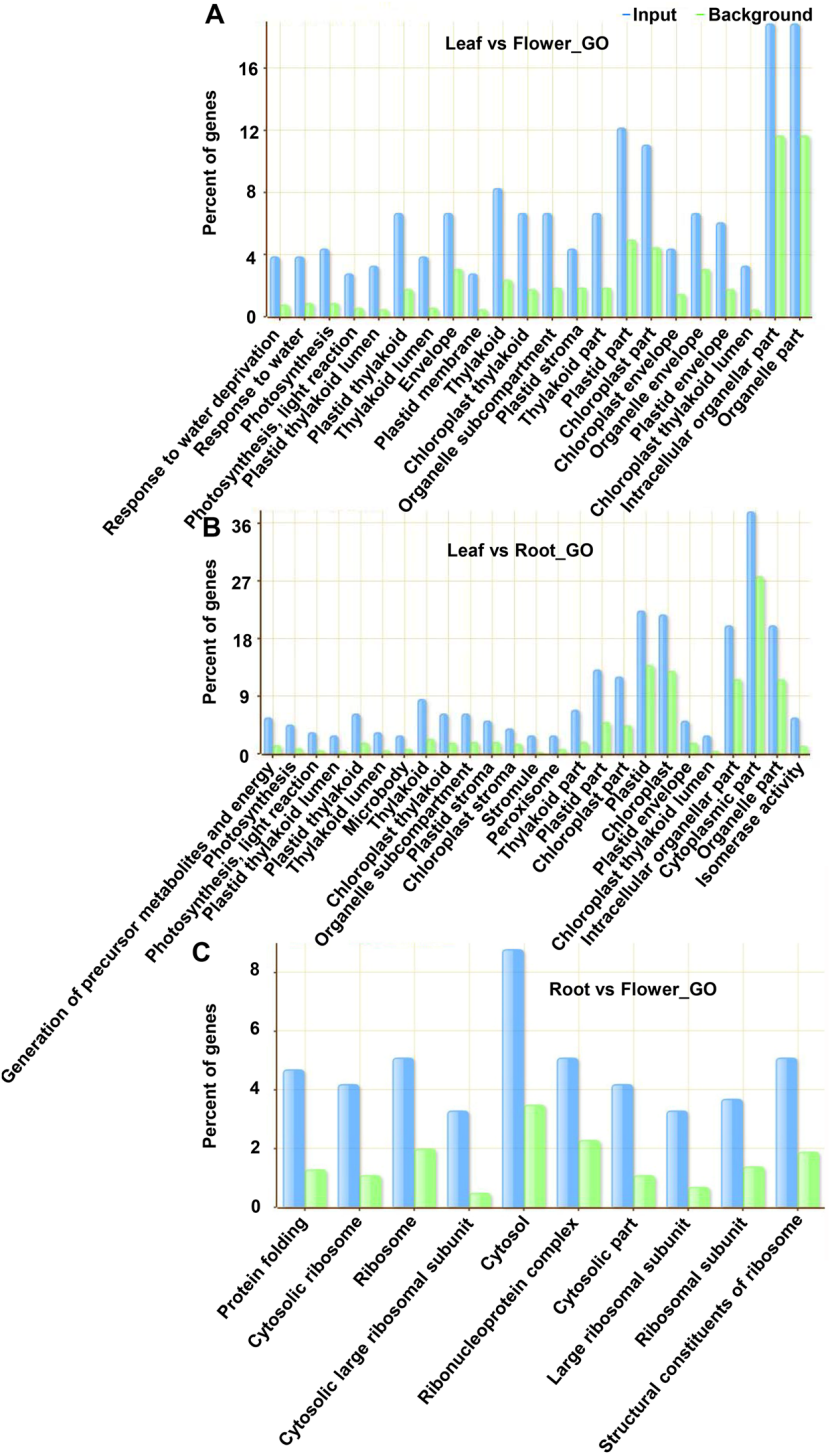
Gene ontology analysis of genes displaying APA between tissue types. (**A–C**) Represent enriched GO categories for the genes displaying APA between leaf-flower, leaf-root, and root-flower, respectively.

Genes displaying tissue-wise differential poly(A) site usage were subjected to Gene Ontology (GO) analysis to elucidate enrichment for specific functional categories. Comparison of poly(A) site usage between leaf and flower tissue identified 323 PACs that mapped to 204 genes and exhibited differential poly(A) site usage (Fig. 4A,B, and Supplemental Dataset S4). GO analysis of this set of genes showed an over-representation of genes involved in photosynthesis, especially in the light reaction of photosynthesis, and genes implicated in responses to water deprivation (Fig. 6A). Similarly, differential poly(A) site usage analysis between leaf and root tissue identified 358 differentially utilized PACs that were in 197 genes (Fig. 4A,B, and Supplemental Dataset S5). GO analysis of this set of genes showed enrichment of genes implicated in the light reaction of photosynthesis, and genes involved in the process of generation of precursor metabolites and energy (Fig. 6B). Finally, the comparison of poly(A) site usage between root and flower tissues identified 390 PACs within 249 genes that showed differential poly(A) site usage (Fig. 4A,B, and Supplemental Dataset S6). GO analysis of this set of genes displayed over-representation of genes involved in ribosome biogenesis, and protein folding (Fig. 6C).

### Tissue-wise expression and APA in genes encoding polyadenylation factors

**Figure 7:**
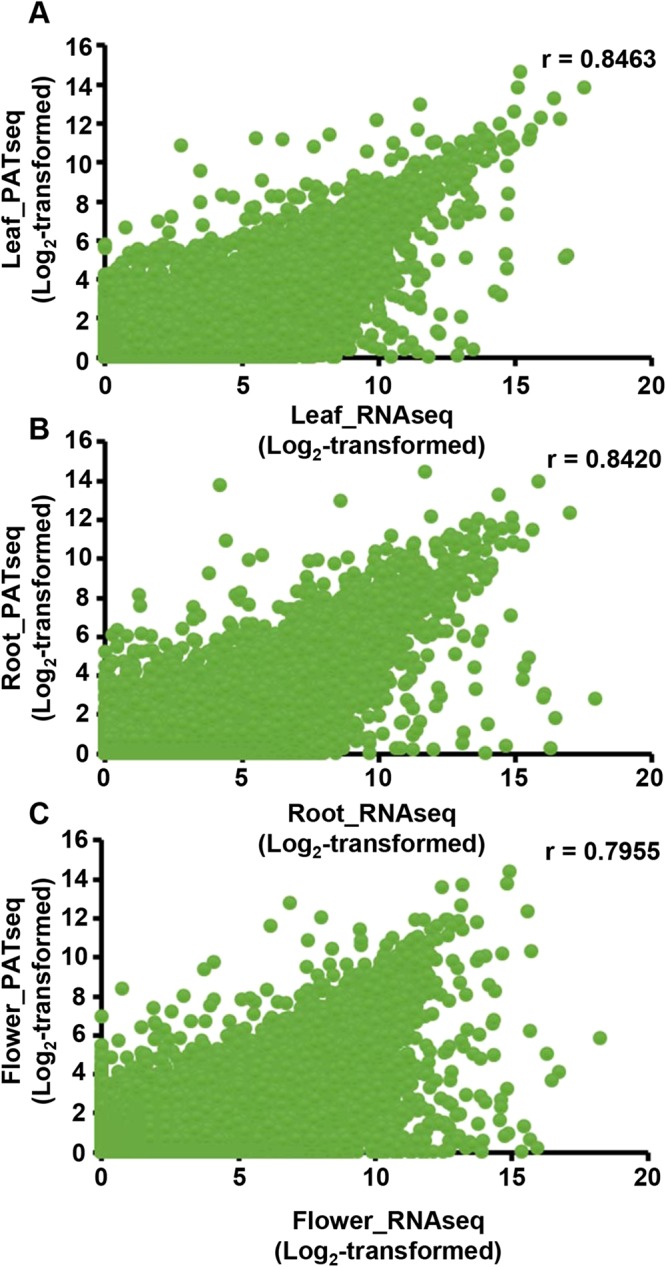
Comparisons of gene expression profiles estimated using PATseq and RNAseq datasets. Gene expression values were normalized, log2-transformed and Pearson correlation coefficients (r) were calculated between gene expression values estimated using PATseq and RNAseq datasets.

Along with providing an account of genome-wide alternative polyadenylation, poly(A) tag sequencing (PATseq) can be a useful and reliable way for analyzing global gene expression^51–54^. Accordingly, we conducted a genome-wide gene expression analysis using PATseq dataset (see Methods). Next, we compared the genome-wide gene expressions profile estimated using the PATseq dataset with gene expression profiles generated using our previously reported RNAseq data (NCBI SRA BioProject accession PRJNA287876). For this comparison RNAseq and PATseq libraries were made from the same set of RNA samples. Pearson correlation coefficients for the gene expressions measurements using PATseq and RNAseq approach in leaf, root, and flower tissue were estimated to be 0.85, 0.84, and 0.80, respectively (Fig. 7). These high correlations between the gene expressions profiles obtained through two approaches suggest that PATseq provides a reasonably reliable account for the global gene expression.

**Figure 8:**
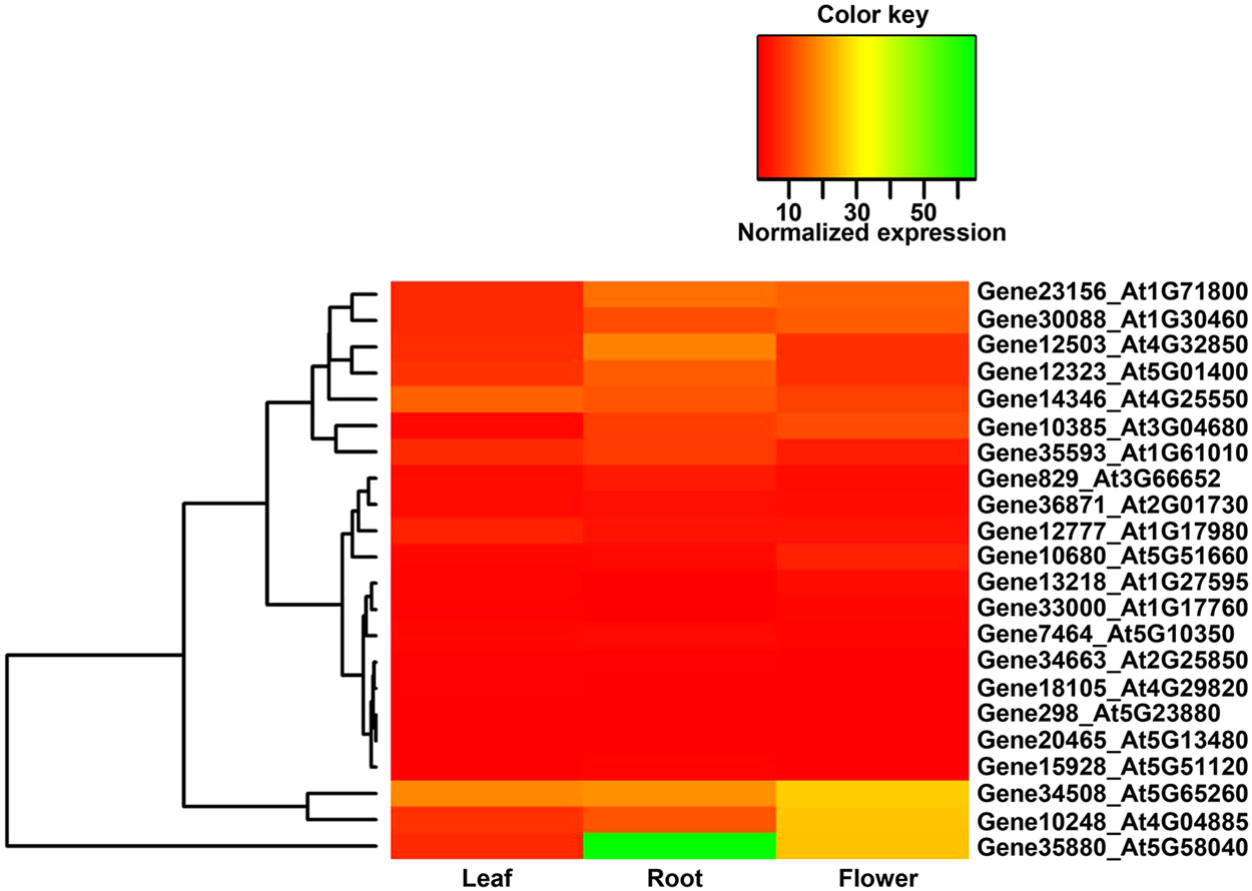
Spatial expressions of genes encoding polyadenylation factors. Red clover genes encoding orthologs of various polyadenylation factors in *Arabidopsis*, were identified using Blast search and their normalized expressions are represented in the heatmap. Scale for the expression values represented in the heatmap is given above.

To further extend our gene expression analysis using poly(A) tags, we assessed the spatial expression of several red clover genes encoding various polyadenylation factors. To this end, we identified orthologs of various polyadenylation factors in red clover, including subunits of CPSF (Cleavage and Polyadenylation Specificity Factor) and CstF (Cleavage Stimulation Factor), poly(A) polymerases, FIP, FY, and others. Next, we analyzed the tissue-wise expression of these genes using the PATseq dataset (Fig. 8). We do not see any significant difference in the tissue-wise expression for the most of the polyadenylation factors. However, the gene encoding FIP1(V) (ortholog for this gene in *Arabidopsis* is *AT5G58040*) displayed significantly higher expression in roots as compared to the other tissues. Moreover, FIP1(V) expression in flowers was also found to be higher than the leaf tissue. Additionally, the red clover gene encoding PCFS4 (PCF11P-similar protein 4, *Arabidopsis* ortholog of which is *AT4G04885*) exhibited maximum expression in flower, followed by lower expression levels in root and leaf tissues, respectively.

**Figure 9:**
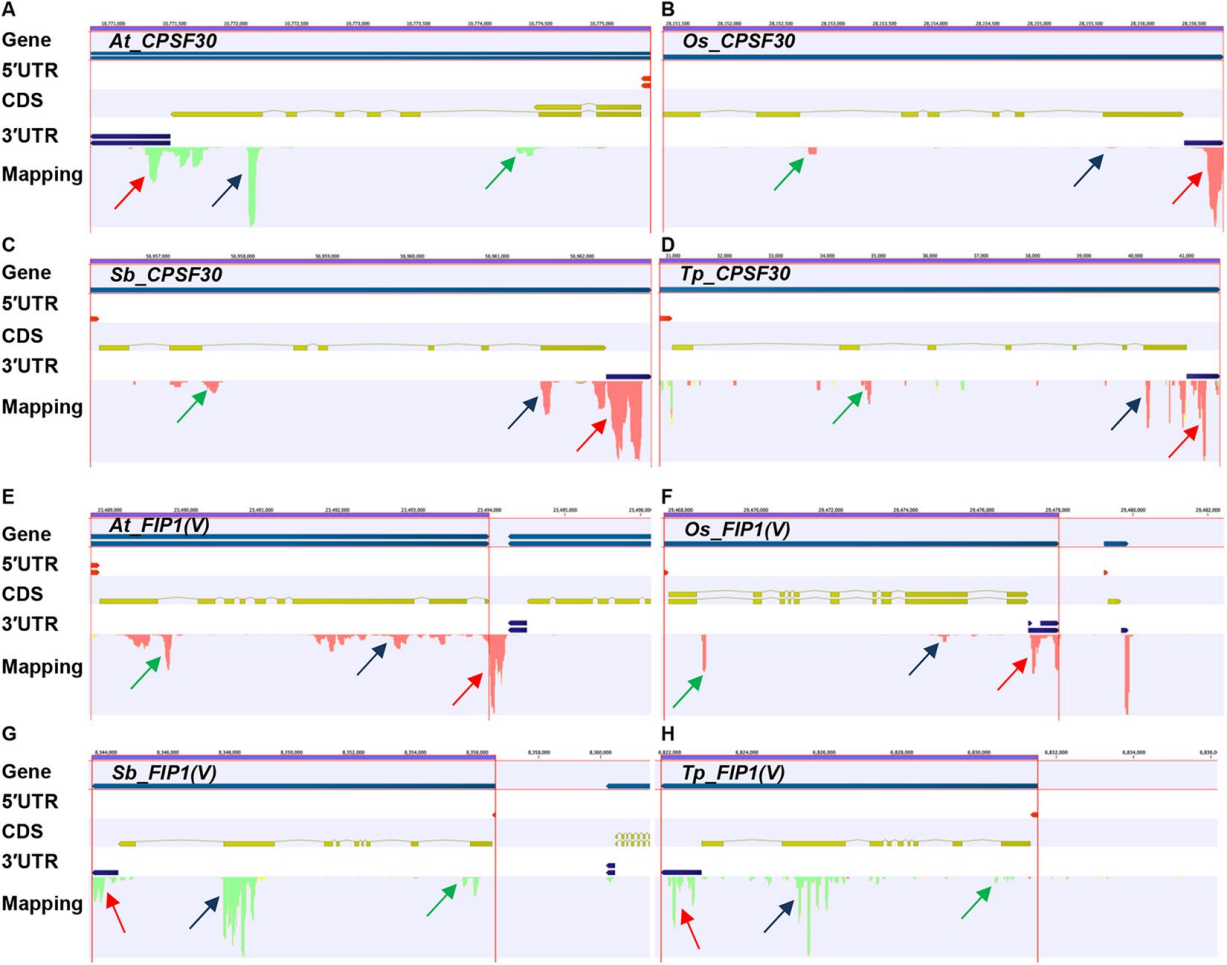
APA in genes encoding polyadenylation factors, CPSF30 and FIP1(V) in different plant species. **(A–D**) Represent read mappings of genes encoding CPSF30 orthologs in arabidopsis, rice, sorghum, and red clover, respectively. **(E–H**) Display read mappings of genes encoding FIP1(V) orthologs in arabidopsis, rice, sorghum, and red clover, respectively. Poly(A) sites located at introns, coding regions and 3′UTRs are represented with green, blue and red arrows, respectively.

Additionally, we explored the evolutionary conservation of APA in the genes encoding polyadenylation factors. Specifically, we have examined the polyadenylation profiles of the genes encoding two key polyadenylation factors, namely CPSF30 (Fig. 9A–D) and FIP1(V) (Fig. 9E–H) in *Arabidopsis*, rice, sorghum, and red clover. Transcripts encoded by the two *Arabidopsis* genes have been shown to be subjects of APA^55,56^. Moreover, APA involving CPSF30-encoding mRNAs is important for nitrate-responsive transcription in at least one gene^57^. Our analysis revealed high degree of conservation of poly(A) site usage for the genes encoding these two polyadenylation factors in both monocot and dicot plant species. In particular, in all four species, the intronic site predicted to yield the so-called CPSF30S protein was seen (Fig. 9A–D, green arrows). A similar intronic event predicted to yield a short FIPV(S) polypeptide was also seen in all four species (Fig. 9E–H, green arrows). There was also considerable conservation in non-canonical CDS-situated APA in these two genes; only in rice were these events relatively rare (Fig. 9A–H, blue arrows). These results reveal a considerable evolutionary conservation of APA in these two genes, and suggest important roles for the various polypeptides, and perhaps as well for contributions of RNA quality control (via the use of CDS sites and subsequent handling of the resulting non-stop mRNAs) in the regulation of expression of these two genes.

## Discussion

### Prevalence of APA and its role in modulating tissue-wise transcriptional dynamics

While there have been reports focusing on transcriptional dynamics in red clover^46,48,58^, there have been none on the role of RNA processing in regulating transcriptome dynamics in this crop. In the current study, we have analyzed the role of alternative polyadenylation in modulating transcriptional dynamics in different tissue types in red clover.

APA can increase transcriptome and proteome diversity through various mechanisms, such as by encoding different transcripts, and protein isoforms, as well as by altering transcript stability through elimination of recognition sites for miRNAs^3,4,59^. Additionally, it is also likely that the usage of non-canonical poly(A) sites located within protein coding regions and introns can direct the transcriptional output to various RNA quality control pathways, and thereby regulate the level of functional transcripts^60–64^. With the rise in the number of genome-wide studies, widespread occurrences of tissue-specific APA have been discovered in several species. There is a wide prevalence of tissue-specific APA events in different mammalian systems^9,40^. In *Caenorhabditis elegans*, tissue-specific APA events were found to be prevalent in intestine, pharynx, and body muscle tissues^11^. Tissue-specific APA was demonstrated between leaf, and seed tissues in *Arabidopsis*^12^. In rice, there is an extensive tissue-wise dynamics in APA profiles^15^. Our current analysis is consistent with all of these studies, especially those done in plants, in that it establishes a substantial prevalence of tissue-wise APA events in the forage legume red clover. Interestingly, proximal poly(A) sites located in the intronic regions were used less in the root tissue as compared to the leaf and flower tissues. In contrast, poly(A) sites located in the protein coding regions displayed preferential usage in the leaf and root tissues as compared to the flower tissue. Such preferential usages of distal and proximal poly(A) sites were reported previously in the neuronal tissue and blood cell and testis in humans^38,41^.

### Individual APA events and their significance in regulating plant developmental processes

Roles of specific APA events in regulating several plant developmental processes have been well documented. Several core polyadenylation factors and RNA-binding proteins were shown to regulate floral initiation. A core polyadenylation factor FY interacts with the RNA-binding protein FCA to promote proximal intronic polyadenylation of the *FCA*-encoded transcripts^29^. Similar to FCA, another RNA-binding protein FPA also undergoes APA and regulates floral initiation^31^. FCA and FPA along with two other core polyadenylation factor subunits CstF64 and CstF77 regulate the enhanced usage of the proximal poly(A) sites of *FLC*-encoded antisense transcripts^30,31^. Similarly, in *Medicago trancatula*, the *SYP132* (*SYNTAXIN132*) gene implicated in legume-rhizobium symbiosis was shown to undergo APA and produce two membrane soluble protein receptors (t-SNARE). Of these two isoforms, SYP132A was found to be induced during symbiosis and essential for maturation of symbiosomes to their functional forms, whereas the SYP132C isoform was shown not to be required for symbiosis, but possesses other important functions^37^. In order to build a better understanding of the biological roles of APA, it is worthwhile to identify such APA events with potential biological implications. Our analysis has identified in total 792 PACs that exhibit APA between two different tissue types in red clover; these PACS affect the expression of 468 genes. The examples illustrated in Fig. 5 suggest interesting contributions of APA towards different developmental processes. For example, red clover gene35272 displayed APA between leaf and root tissues (Fig. 5B), with the functional mRNA isoform derived from distal poly(A) site choice being preferentially expressed in roots. Specifically, in leaf tissue, the red clover gene35272-encoded transcripts predominantly use a non-canonical poly(A) site, whereas, in the root tissue, the canonical 3′UTR site was used mostly (Fig. 5B). It is most likely that this gene generates full length functional transcripts mostly in the root tissue. This gene is orthologous to the Arabidopsis gene *At4G13270*, which encodes a LEA (Late Embryogenesis Abundant) protein. While LEA proteins are mostly implicated in seed development and mediating responses to environmental stresses^65^, a gene encoding SAG21/AtLEA5 was reported to be involved in root development and mediating biotic stress response^66^. It would be interesting in future to test if this APA event has a role in regulating root development under normal physiological condition or environmental stresses.

Another red clover gene (Gene24502) exhibited APA between leaf and flower tissues (Fig. 5C). This gene is orthologous to the Arabidopsis gene *At4G27790*, which encodes a calcium-binding EF-hand family protein. Calcium-binding proteins are well known for their roles in hormonal regulation and in mediating responses to environmental stresses^67^. EF-hand family proteins possess a conserved helix-loop-helix structure that can bind to a single Ca^2+^ ion^68^. EF-hand family proteins were implicated in environmental and nutritional stress signaling in soybean^69^. In the leaf tissue, the red clover gene24502 predominantly used a non-canonical poly(A) site located at the 5′UTR. In contrast, in the flower tissue, this gene mostly used the canonical poly(A) site located at the 3′UTR (Fig. 5C). This observation suggests a flower-specific expression and role for gene24502 in floral development, and provides a possible conceptual link between flower development and calcium sensing and signaling.

### Possible mechanisms of regulation of spatial APA in red clover

The changes in the usage of different classes of poly(A) sites may arise due to various reasons. One possible reason behind the preferred usage of certain classes of poly(A) sites may be alterations in the activities of core polyadenylation complex mediated by plethora of RNA-binding proteins. It has been shown previously in the model plant *Arabidopsis*, that two RNA-binding proteins, FPA and FCA played significant role in the poly(A) site choice^29,70,71^. Our analysis has revealed that nucleotide profiles around poly(A) sites located in different genomic regions closely resemble patterns previously observed in other plant species, including *Arabidopsis*, *Medicago* and rice (Fig. 3)^12,13,15^. This indicates a high degree of conservation of poly(A) signals in different tissue types. This finding suggests that instead of a global regulation through RNA-protein interactions, there may be contribution of specific RNA-protein interactions in mediating spatial APA in red clover. However, inclusion of more tissue types in future experiments may unearth novel tissue-specific poly(A) signals. Such expectations are not farfetched considering the recent discovery in rice of a T-rich motif in the poly(A) sites mapped to the intronic regions in pollen as compared to the A-rich motif in the same region in leaf^15^.

It has been shown both in mammals and in plants that alterations in various polyadenylation factors resulted in significant changes in poly(A) site choice. These studies demonstrated the roles of Poly(A) Binding Protein Nuclear 1 (PABPN1), Cleavage Factor I (CF I), Cleavage and Polyadenylation Specificity Factor 30 (CPSF30), CPSF100, Fip1, and CstF64 in poly(A) site choice^49,72–76^. Additionally, it was also shown that cleavage and polyadenylation factors Pcf11 and Fip1 promote the usage of proximal poly(A) sites, whereas CFI-25/68, PABPN1, and PABPC1 enhance the usage of distal poly(A) sites^77^. Our results have displayed significantly dynamic spatial expressions of two polyadenylation factors (Fig. 8). The gene encoding ortholog of FIP1(V) expressed at significantly higher level in root as compared to other tissue types tested. FIP1(V) is a RNA-binding protein and considered as a part of the CPSF complex. FIP1(V) was shown to interact with several polyadenylation factors and to directly affect activity of poly(A) polymerase^78,79^. We have also detected higher expression of the gene encoding PCFS4 in flower as compared to the other tissues (Fig. 8). In *Arabidopsis*, PCFS4 was reported to regulate alternative polyadenylation of FCA and promote flowering^80^. These results suggest that FIP1(V) and PCFS4 may be involved in mediating spatial APA in red clover. Some of the polyadenylation factor-encoding genes produce multiple isoforms, such as *CstF64* in mammals and *CPSF30* in plants^55,81,82^. It would be interesting in future to test differential expressions of two isoforms of these factors under different development and physiological conditions and to assess if two isoforms differentially regulate poly(A) site choice under such conditions. Together with our previously reporter spatial gene expression profile, this genome-wide spatial alternative polyadenylation study will provide an account of global spatial transcriptional dynamics in the non-model forage legume red clover.

## Methods

### Plant materials and growth condition

Three individual lines of red clover cultivar ‘Kenland’ were grown under greenhouse conditions with 16/8 h of light/dark cycle. At the 7–8 leaf stage, clones of individual lines were separated and three clones for each line were transplanted. For the RNA extraction, leaf, root and flower tissues were collected in triplicates. For each replicate, tissue samples from three clones of each line were pooled. Root and leaf tissues were collected two weeks post transplantation. Flower tissues were collected after a plant bears several flowers. All tissue samplings were conducted between 10 AM to noon to avoid any diurnal variations. Tissue samples were flash frozen in liquid nitrogen as collected and stored at −80 °C until further use.

### RNA extraction, PATseq library preparation and quality assessment

RNA extraction was performed using Trizol® reagent (Life Technologies) as per the instructions of the manufacturers. Following isolation, RNA samples were purified with RNA mini spin column (Enzymax LLC). 1 µg of purified total RNA was used to make PATseq libraries using a modified version of a previously published protocol^83^. Briefly, total RNA was fragmented, followed by poly(A) enrichment with oligo dT beads. Next, 3′end fragments of poly(A) enriched RNA were reverse transcribed. Primers for reverse transcription consist of unique barcodes for multiplexing, a sequencing adapter for the Illumina platform, and a T_18_VN sequence at the 3′ end. Reverse transcription reactions were followed by strand-switching using so-called SMART technology (Clontech Laboratories, Inc.). Second sequencing adapter was added to the SMART oligonucleotide. Following reverse transcription and strand-switching, two rounds of size selection were performed using AMPure beads (Agencourt AMPure XP beads, Beckman Coulters, Inc.). Next, cDNAs were PCR amplified, and further size selected using gel purification. Gel purified products were re-amplified, and subjected to one round of purification with the SPRI beads to get the final library. Quality of the PATseq libraries was checked using Agilent High sensitivity DNA chips (Agilent Technologies). PATseq libraries were quantified using Qubit® fluorometer with Qubit® dsDNA HS assay kit (Life Technologies).

### High-throughput sequencing and data processing

PATseq libraries were sequenced using Illumina platform (HiSeq 2500, 1 × 76 nucleotides). After retrieval of the sequences in fastq format, raw reads were demultiplexed, and adapter, barcode sequences, and poly(T) tracts were trimmed using CLC Genomics Workbench. Ribosomal RNA (rRNA) sequences were removed by mapping the processed reads to *Arabidopsis thaliana* rRNA sequences. Processed reads were then mapped to the red clover genome sequence (redclover_v2.1, https://zenodo.org/record/17232)^47^. For mapping red clover PATseq reads following parameters were used: match score-1, mismatch cost-2, cost of insertions and deletions- linear gap cost, insertion cost-3, deletion cost-3, length fraction-0.9 and similarity fraction-0.8. For the APA analysis, 3′UTRs in the redclover_v2.1 annotation were extended by 200 nucleotides in the 3′ direction and the modified annotated genome used as reference for the mapping of processed reads. The decision to extend 3′UTRs was arrived at empirically, by mapping reads to modified genomes with 3′ extensions of 100–400 bp in 100 bp increments, and choosing the point at the numbers of mapped reads ceased to increase. While mapping, genomic regions with eight or more consecutive ‘A’s were masked to eliminate reads generated due to possible internal priming during the reverse transcription reaction.

### Genome-wide analysis of alternative polyadenylation

The mapping results were exported from the CLC Genomics Workbench in ‘bam’ file format. Further analyses were conducted using BEDTools. Briefly, ‘bam’ files were converted to ‘bed’ file format, followed by tag trimming to reduce each sequence tag to a single nucleotide; the trimmed tags were then sorted for subsequent processing. Next, a list of poly(A) clusters (PACs) were prepared, where poly(A) sites (PASs) within 24 nucleotides from one another were grouped in one PAC as described previously^12^. For the subsequent analysis, PACs defined by 20 or more PATs in the complete dataset were reserved, and the other PACs discarded. A list of PASs was also generated, and PASs defined by 8 or more PATs in the complete dataset were retained for further analysis. Next, to each PAC and PAS, corresponding gene IDs, and genomic regions were added. Global distributions of PACs and PASs across different genomic regions were calculated. Additionally, the distributions of PATs mapping to various genomic locations were estimated for each tissue type. The numbers of mapped PATs were calculated for each PAC, and each gene. Relative poly(A) site usages were calculated by dividing number of PATs mapping to a PAC by the total number of PATs that mapped to the associated gene. Differences in relative poly(A) site usage between different tissue types were estimated by subtracting relative poly(A) site usage of a PAC in one tissue type from the relative poly(A) site usage of the same PAC in another tissue type. Such differences were calculated and the results recorded in spreadsheets and displayed with boxplots.

### Validation of PACs using an independent RNAseq dataset

PACs generated using the PATseq dataset were validated using a red clover RNAseq dataset [NCBI Short Read Archive (SRA) BioProject accession PRJNA287846]^48^. Briefly, poly(A) tail containing reads were extracted from the RNAseq dataset and were mapped to the red clover genome using the same criteria as described above. A PAC list was generated as described in the preceding section and PACs with at least two PATs were retained for further analysis. Overlaps between the genomic coordinated of PACs in two datasets were assessed using BEDTools.

### Nucleotide composition analysis

From the master PAS list, separate lists of PASs were generated for leaf, root and flower samples. For each sample, number of PAT for each PAS, was normalized by the total number of PATs in all PASs, and expressed in millions. Next, averages of normalized PATs were calculated for three replicates of each tissue sample. Only PASs with ≥2 normalized PATs were retained for the nucleotide composition analysis. Analysis was done by mapping three replicates of each sample together. Genomic regions and corresponding genes were added to each PAS, and PASs were grouped according to their genomic locations. PASs with no corresponding genes were removed from the subsequent analysis. Next, sequences spanning 100 bp upstream to 100 bp downstream of PASs were extracted, and any sequences less than 201 bp in length were removed from the analysis. For each class of PASs (those located in 3′UTR, protein coding region, intron, and 5′UTR), the proportion of each of the four bases was calculated for each position relative to the poly(A) site (+1). To analyze nucleotide profiles of the sequences surrounding sites, where internal priming may happen, co-ordinates of such sites (these sites are defined by ‘N6A8’ motif, where ‘N’ is any nucleotide) were extracted, extended by 100 nucleotides in both directions and grouped according to their genomic locations. Nucleotide profiles of sequences encompassing potential internal priming sites located at 3′UTR and coding regions in the red clover genome, were estimated as stated before.

### Identification of PACs and genes displaying APA

Differentially utilized PACs between two tissue types were determined using DEXseq software implemented in R^84^. Briefly, ‘bed’ files representing mappings of trimmed PATs to the red clover genome were converted to ‘bam’ format using BEDTools. SAMTools was used to convert these ‘bam’ files to ‘sam’ format. A ‘GTF’ file was generated from the PAC list with the gene annotation. A python script was used to convert this ‘GTF’ file to ‘GFF’ format suitable for use by DEXseq. Additionally, another python script was used to count the number of reads in each PAC in each library and outputs from this step was saved in ‘txt’ format and were used for DEXseq analysis in R. Statistical analyses in DEXseq were performed with an adjusted p value of <0.05 to identify PACs showing differential poly(A) site usage between two tissue types. Genes associated with the differentially utilized PACs between two tissue samples were subjected to gene ontology (GO) analysis using ‘Singular Enrichment Analysis’ (SEA) tool in AgriGO^85^. Enriched GO categories were identified using ‘chi-square test’ using following parameters: Hochberg (FDR) cut-off value 0.1 and minimum number of mapping entries of 5.

### Comparison of gene expression using PATseq and RNAseq

To analyze the usefulness of PATseq data in quantifying global gene expression, we conducted gene expression analyses with both RNAseq and PATseq datasets and compared results from two analyses. For the gene expression analysis using RNAseq approach, our previously reported RNAseq dataset was used [NCBI Short Read Archive (SRA) BioProject accession PRJNA287846 (https://www.ncbi.nlm.nih.gov/bioproject/PRJNA287846/)]. The gene expression analysis using RNAseq dataset was performed in ‘CLC Genomics Workbench’ using ‘RNAseq’ suite. To quantify gene expression using PATseq approach, the PAT frequency for each gene was calculated using BEDTools and the output file was imported in ‘CLC Genomics Workbench’ for the subsequent analysis. In both cases, expression values were transformed by adding 1, and then normalized using ‘quantile normalization’. Normalized gene expression values of RNAseq and PATseq datasets were log2-transformed and Pearson correlation coefficients between two datasets were calculated.

### Data availability

High throughput sequence data generated in this study was deposited to the NCBI SRA under the BioProject accession PRJNA412508.

## Electronic supplementary material

Supplementary figures and tables

Supplementary datasets

## References

[1] Marzluff WF, Wagner EJ, Duronio RJ (2008). Metabolism and regulation of canonical histone mRNAs: life without a poly(A) tail. Nat Rev Genet.

[2] Neve J, Furger A (2014). Alternative polyadenylation: less than meets the eye?. Biochemical Society Transactions.

[3] Tian B, Manley JL (2017). Alternative polyadenylation of mRNA precursors. Nat Rev Mol Cell Biol.

[4] Elkon R, Ugalde AP, Agami R (2013). Alternative cleavage and polyadenylation: extent, regulation and function. Nat Rev Genet.

[5] Lutz CS, Moreira A (2011). Alternative mRNA polyadenylation in eukaryotes: an effective regulator of gene expression. Wiley Interdiscip Rev RNA.

[6] Gautheret D, Poirot O, Lopez F, Audic S, Claverie JM (1998). Alternate polyadenylation in human mRNAs: a large-scale analysis by EST clustering. Genome Res.

[7] Tian B, Hu J, Zhang HB, Lutz CS (2005). A large-scale analysis of mRNA polyadenylation of human and mouse genes. Nucleic Acids Research.

[8] Miura P, Shenker S, Andreu-Agullo C, Westholm JO, Lai EC (2013). Widespread and extensive lengthening of 3′ UTRs in the mammalian brain. Genome Research.

[9] Derti A (2012). A quantitative atlas of polyadenylation in five mammals. Genome Research.

[10] Ozsolak F (2010). Comprehensive Polyadenylation Site Maps in Yeast and Human Reveal Pervasive Alternative Polyadenylation. Cell.

[11] Blazie SM (2015). Comparative RNA-Seq analysis reveals pervasive tissue-specific alternative polyadenylation in Caenorhabditis elegans intestine and muscles. BMC Biol.

[12] Wu XH (2011). Genome-wide landscape of polyadenylation in Arabidopsis provides evidence for extensive alternative polyadenylation. Proceedings of the National Academy of Sciences of the United States of America.

[13] Wu, X., Gaffney, B., Hunt, A. G. & Li, Q. Q. Genome-wide determination of poly(A) sites in Medicago truncatula: evolutionary conservation of alternative poly(A) site choice. *Bmc Genomics* **15**, 10.1186/1471-2164-15-615 (2014). 10.1186/1471-2164-15-615 PMC4117952 25048171

[14] Shen Y (2008). Genome level analysis of rice mRNA 3′-end processing signals and alternative polyadenylation. Nucleic Acids Res.

[15] Fu H (2016). Genome-wide dynamics of alternative polyadenylation in rice. Genome Res.

[16] Sherstnev A (2012). Direct sequencing of Arabidopsis thaliana RNA reveals patterns of cleavage and polyadenylation. Nature Structural & Molecular Biology.

[17] Higgs DR (1983). Alpha-Thalassemia Caused By A Polyadenylation Signal Mutation. Nature.

[18] Orkin SH, Cheng TC, Antonarakis SE, Kazazian HH (1985). Thalassemia Due To A Mutation In The Cleavage-Polyadenylation Signal Of The Human Beta-Globin Gene. Embo Journal.

[19] Gehring NH (2001). Increased efficiency of mRNA 3′ end formation: a new genetic mechanism contributing to hereditary thrombophilia. Nature Genetics.

[20] Jenal M (2012). The poly(A)-binding protein nuclear 1 suppresses alternative cleavage and polyadenylation sites. Cell.

[21] Bennett CL (2001). A rare polyadenylation signal mutation of the FOXP3 gene (AAUAAA −>AAUGAA) leads to the IPEX syndrome. Immunogenetics.

[22] Gieselmann V, Polten A, Kreysing J, von Figura K (1989). Arylsulfatase A pseudodeficiency: loss of a polyadenylylation signal and N-glycosylation site. Proc Natl Acad Sci USA.

[23] Barth ML, Fensom A, Harris A (1993). Prevalence of common mutations in the arylsulphatase A gene in metachromatic leukodystrophy patients diagnosed in Britain. Hum Genet.

[24] Thomas CP, Andrews JI, Liu KZ (2007). Intronic polyadenylation signal sequences and alternate splicing generate human soluble Flt1 variants and regulate the abundance of soluble Flt1 in the placenta. FASEB J.

[25] Thomas CP, Raikwar NS, Kelley EA, Liu KZ (2010). Alternate processing of Flt1 transcripts is directed by conserved cis-elements within an intronic region of FLT1 that reciprocally regulates splicing and polyadenylation. Nucleic Acids Res.

[26] Ashar-Patel A (2017). FLT1 and transcriptome-wide polyadenylation site (PAS) analysis in preeclampsia. Sci Rep.

[27] Park JY (2011). Comparative analysis of mRNA isoform expression in cardiac hypertrophy and development reveals multiple post-transcriptional regulatory modules. PLoS One.

[28] Mayr C, Bartel DP (2009). Widespread Shortening of 3′ UTRs by Alternative Cleavage and Polyadenylation Activates Oncogenes in Cancer Cells. Cell.

[29] Simpson GG, Dijkwel PP, Quesada V, Henderson I, Dean C (2003). FY is an RNA 3′ end-processing factor that interacts with FCA to control the Arabidopsis floral transition. Cell.

[30] Liu F, Marquardt S, Lister C, Swiezewski S, Dean C (2010). Targeted 3′ processing of antisense transcripts triggers Arabidopsis FLC chromatin silencing. Science.

[31] Hornyik C, Terzi LC, Simpson GG (2010). The spen family protein FPA controls alternative cleavage and polyadenylation of RNA. Dev Cell.

[32] Giranton JL, Ariza MJ, Dumas C, Cock JM, Gaude T (1995). The S locus receptor kinase gene encodes a soluble glycoprotein corresponding to the SKR extracellular domain in Brassica oleracea. Plant J.

[33] Tantikanjana T, Nasrallah ME, Stein JC, Chen CH, Nasrallah JB (1993). An alternative transcript of the S locus glycoprotein gene in a class II pollen-recessive self-incompatibility haplotype of Brassica oleracea encodes a membrane-anchored protein. Plant Cell.

[34] Tang G (2002). The bifunctional LKR/SDH locus of plants also encodes a highly active monofunctional lysine-ketoglutarate reductase using a polyadenylation signal located within an intron. Plant Physiol.

[35] Cyrek M (2016). Seed Dormancy in Arabidopsis Is Controlled by Alternative Polyadenylation of DOG1. Plant Physiol.

[36] Fedak H (2016). Control of seed dormancy in Arabidopsis by a cis-acting noncoding antisense transcript. Proc Natl Acad Sci USA.

[37] Pan H (2016). A symbiotic SNARE protein generated by alternative termination of transcription. Nat Plants.

[38] Zhang H, Lee JY, Tian B (2005). Biased alternative polyadenylation in human tissues. Genome Biol.

[39] Smibert P (2012). Global patterns of tissue-specific alternative polyadenylation in Drosophila. Cell Rep.

[40] Lianoglou S, Garg V, Yang JL, Leslie CS, Mayr C (2013). Ubiquitously transcribed genes use alternative polyadenylation to achieve tissue-specific expression. Genes & Development.

[41] Liu DL (2007). Systematic variation in mRNA 3′-processing signals during mouse spermatogenesis. Nucleic Acids Research.

[42] Shen YJ (2011). Transcriptome dynamics through alternative polyadenylation in developmental and environmental responses in plants revealed by deep sequencing. Genome Research.

[43] Frame, J., Charlton, J. F. L. & Laidlaw, A. S. Temperate Forage Legumes. *Wallingford: CAB International* (1998).

[44] Taylor, N. L. In *Clover Science and Technology*. (ed. Norman. L. Taylor) 1–6. (American Society of Agronomy, Inc., Crop Science Society of America, Inc., Soil Science Society of America, Inc., 1985).

[45] Taylor NL (2008). A century of clover breeding developments in the United States. Crop Science.

[46] Yates, S. A. *et al*. *De novo* assembly of red clover transcriptome based on RNA-Seq data provides insight into drought response, gene discovery and marker identification. *BMC Genomics* **15**, (9 June 2014)-(2019 June 2014) (2014). 10.1186/1471-2164-15-453 PMC4144119 24912738

[47] De Vega JJ (2015). Red clover (Trifolium pratense L.) draft genome provides a platform for trait improvement. Sci Rep.

[48] Chakrabarti, M., Dinkins, R. D. & Hunt, A. G. *De novo* Transcriptome Assembly and Dynamic Spatial Gene Expression Analysis in Red Clover. *Plant Genome* **9**, 10.3835/plantgenome2015.06.0048 (2016). 10.3835/plantgenome2015.06.0048 27898811

[49] Thomas PE (2012). Genome-Wide Control of Polyadenylation Site Choice by CPSF30 in Arabidopsis. Plant Cell.

[50] Loke JC (2005). Compilation of mRNA polyadenylation signals in Arabidopsis revealed a new signal element and potential secondary structures. Plant Physiol.

[51] Sigurgeirsson B, Emanuelsson O, Lundeberg J (2014). Sequencing degraded RNA addressed by 3′ tag counting. PLoS One.

[52] Beck AH (2010). 3′-end sequencing for expression quantification (3SEQ) from archival tumor samples. PLoS One.

[53] Brunner AL (2012). Transcriptional profiling of long non-coding RNAs and novel transcribed regions across a diverse panel of archived human cancers. Genome Biol.

[54] Moll, P., Ante, M., Seitz, A. & Reda, T. QuantSeq. 3′ mRNA sequencing for RNA quantification. *Nature Methods* **11**, 10.1038/nmeth.f.376 (2014).

[55] Delaney KJ (2006). Calmodulin interacts with and regulates the RNA-binding activity of an Arabidopsis polyadenylation factor subunit. Plant Physiology.

[56] Hunt, A. G., Xing, D. & Li, Q. Q. Plant polyadenylation factors: conservation and variety in the polyadenylation complex in plants. *Bmc Genomics* **13**, 10.1186/1471-2164-13-641 (2012). 10.1186/1471-2164-13-641 PMC3538716 23167306

[57] Li Z (2017). The Arabidopsis CPSF30-L gene plays an essential role in nitrate signaling and regulates the nitrate transceptor gene NRT1.1. New Phytol.

[58] Kovi MR, Amdahl H, Alsheikh M, Rognli OA (2017). *De novo* and reference transcriptome assembly of transcripts expressed during flowering provide insight into seed setting in tetraploid red clover. Sci Rep.

[59] Tian B, Manley JL (2013). Alternative cleavage and polyadenylation: the long and short of it. Trends Biochem Sci.

[60] de Lorenzo L, Sorenson R, Bailey-Serres J, Hunt AG (2017). Noncanonical Alternative Polyadenylation Contributes to Gene Regulation in Response to Hypoxia. Plant Cell.

[61] Drechsel G (2013). Nonsense-mediated decay of alternative precursor mRNA splicing variants is a major determinant of the Arabidopsis steady state transcriptome. Plant Cell.

[62] Frischmeyer PA (2002). An mRNA surveillance mechanism that eliminates transcripts lacking termination codons. Science.

[63] Shaul O (2015). Unique Aspects of Plant Nonsense-Mediated mRNA Decay. Trends Plant Sci.

[64] van Hoof A, Frischmeyer PA, Dietz HC, Parker R (2002). Exosome-mediated recognition and degradation of mRNAs lacking a termination codon. Science.

[65] Hundertmark, M. & Hincha, D. K. LEA (Late Embryogenesis Abundant) proteins and their encoding genes in Arabidopsis thaliana. *Bmc Genomics* **9**, 10.1186/1471-2164-9-118 (2008). 10.1186/1471-2164-9-118 PMC2292704 18318901

[66] Salleh FM (2012). A novel function for a redox-related LEA protein (SAG21/AtLEA5) in root development and biotic stress responses. Plant Cell and Environment.

[67] Sarwat M, Ahmad P, Nabi G, Hu X (2013). Ca2+ signals: The versatile decoders of environmental cues. Critical Reviews in Biotechnology.

[68] Day, I. S., Reddy, V. S., Ali, G. S. & Reddy, A. S. N. Analysis of EF-hand-containing proteins in Arabidopsis. *Genome Biology* **3** (2002). 10.1186/gb-2002-3-10-research0056 PMC134623 12372144

[69] Zeng, H. Q., Zhang, Y. X., Zhang, X. J., Pi, E. X. & Zhu, Y. Y. Analysis of EF-Hand Proteins in Soybean Genome Suggests Their Potential Roles in Environmental and Nutritional Stress Signaling. *Frontiers in Plant Science* **8**, 10.3389/fpls.2017.00877 (2017). 10.3389/fpls.2017.00877 PMC5443154 28596783

[70] Duc C, Sherstnev A, Cole C, Barton GJ, Simpson GG (2013). Transcription termination and chimeric RNA formation controlled by Arabidopsis thaliana FPA. PLoS Genet.

[71] Lyons R (2013). The RNA-binding protein FPA regulates flg22-triggered defense responses and transcription factor activity by alternative polyadenylation. Sci Rep.

[72] de Klerk E (2012). Poly(A) binding protein nuclear 1 levels affect alternative polyadenylation. Nucleic Acids Res.

[73] Martin G, Gruber AR, Keller W, Zavolan M (2012). Genome-wide analysis of pre-mRNA 3′ end processing reveals a decisive role of human cleavage factor I in the regulation of 3′ UTR length. Cell Rep.

[74] Lackford B (2014). Fip1 regulates mRNA alternative polyadenylation to promote stem cell self-renewal. Embo Journal.

[75] Yao CG (2012). Transcriptome-wide analyses of CstF64-RNA interactions in global regulation of mRNA alternative polyadenylation. Proceedings of the National Academy of Sciences of the United States of America.

[76] Lin JC, Xu RW, Wu XH, Shen YJ, Li QSQ (2017). Role of cleavage and polyadenylation specificity factor 100: anchoring poly(A) sites and modulating transcription termination. Plant Journal.

[77] Li W (2015). Systematic profiling of poly(A)+ transcripts modulated by core 3′ end processing and splicing factors reveals regulatory rules of alternative cleavage and polyadenylation. PLoS Genet.

[78] Hunt AG (2008). Arabidopsis mRNA polyadenylation machinery: comprehensive analysis of protein-protein interactions and gene expression profiling. Bmc Genomics.

[79] Forbes KP, Addepalli B, Hunt AG (2006). An Arabidopsis Fip1 homolog interacts with RNA and provides conceptual links with a number of other polyadenylation factor subunits. Journal of Biological Chemistry.

[80] Xing DH, Zhao HW, Xu RQ, Li QSQ (2008). Arabidopsis PCFS4, a homologue of yeast polyadenylation factor Pcf11p, regulates FCA alternative processing and promotes flowering time. Plant Journal.

[81] Wallace AM (1999). Two distinct forms of the 64,000 Mr protein of the cleavage stimulation factor are expressed in mouse male germ cells. Proc Natl Acad Sci USA.

[82] Shankarling GS, Coates PW, Dass B, Macdonald CC (2009). A family of splice variants of CstF-64 expressed in vertebrate nervous systems. BMC Mol Biol.

[83] Ma L, Pati PK, Liu M, Li QQ, Hunt AG (2014). High throughput characterizations of poly(A) site choice in plants. Methods.

[84] Anders S, Reyes A, Huber W (2012). Detecting differential usage of exons from RNA-seq data. Genome Res.

[85] Du Z, Zhou X, Ling Y, Zhang Z, Su Z (2010). agriGO: a GO analysis toolkit for the agricultural community. Nucleic Acids Res.

