# Supplementary material for: Genome-wide atlas of alternative polyadenylation in the forage legume red clover
Source: Sci Rep. 2018 Jul 27;8:11379. doi: 10.1038/s41598-018-29699-7 (PMC6063945; doi:10.1038/s41598-018-29699-7)
Supplement: Supplementary file 1 — Supplementary figures and tables [file 41598_2018_29699_MOESM1_ESM.pdf]

Genome-wide atlas of alternative polyadenylation in the forage legume red clover

Manohar Chakrabarti[1], Randy D. Dinkins[1,2] and Arthur G. Hunt[1,*]

[1]Department of Plant and Soil Sciences, University of Kentucky, Lexington, KY, USA. 40546-0312.

[2]USDA-ARS, Forage-Animal Production Research Unit, Lexington, Kentucky, USA. 40546.

[*]Corresponding author: A.G.H (aghunt00@uky.edu)

Supplementary Information

Supplemental Fig.S1. Pair-wise comparison of poly(A) metric between replicates of each tissue sample.

Supplemental Fig.S2. Correlation scatterplot depicting relationships between different tissue samples and replicates of each tissue type.

Supplemental Fig.S3. Nucleotide composition of the sequences surrounding potential internal priming sites.

Supplemental Table.S1. Mapping statistics of PATseq libraries.

Supplemental Table.S2. Statistics of PATs extracted from the RNAseq libraries.

Supplemental Table.S3. Pearson correlation coefficients between replicates of PATseq libraries.

Supplemental Dataset.S1. List of poly(A) sites.

Supplemental Dataset.S2. List of poly(A) clusters.

Supplemental Dataset.S3. List of PACs validated with an independent RNAseq dataset.

Supplemental Dataset.S4. PACs and genes displaying APA between leaf and flower.

Supplemental Dataset.S5. PACs and genes displaying APA between leaf and root.

Supplemental Dataset.S6. PACs and genes displaying APA between root and flower.

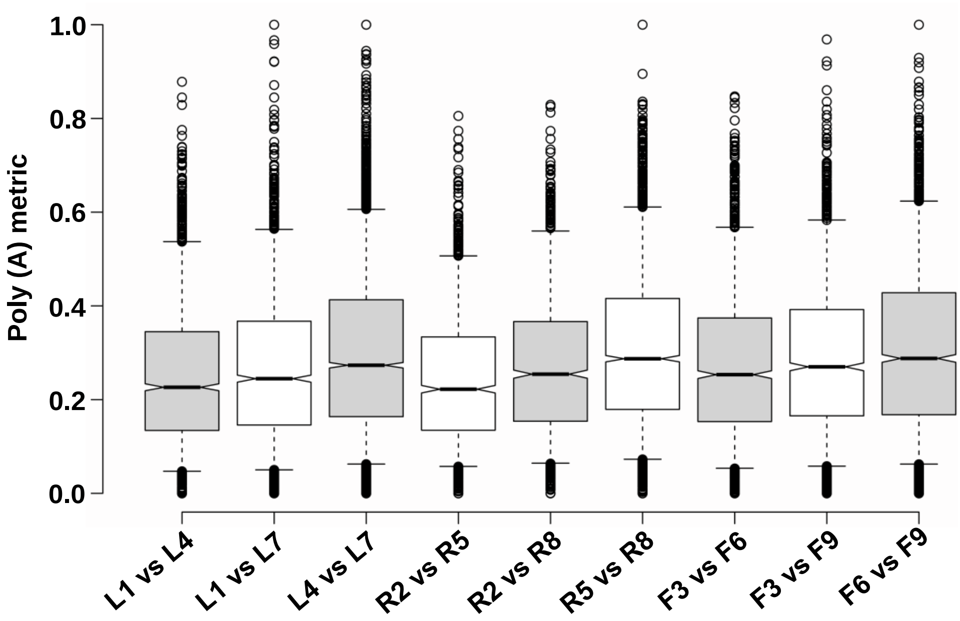

Supplemental Fig.S1. Pair-wise comparison of poly(A) metric between replicates of each tissue sample. Poly(A) metric was calculated as described previously (Thomas et al., 2012). Poly(A) metric of 0 and 1 represent absolute similarity and dissimilarity between two replicates. L, R, and F represent leaf, root, and flower tissue, respectively. 1 to 9 represent sample IDs.

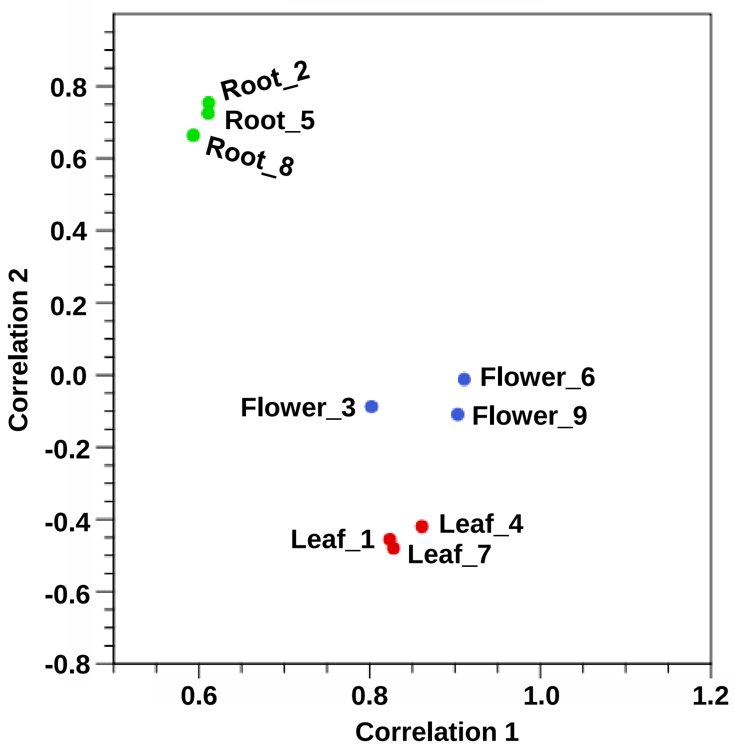

Supplemental Fig.S2. Correlation scatterplot depicting relationships between different tissue samples and replicates of each tissue type. Three biological replicates were used for each tissue type. Correlation scatterplot was generated based on the normalized expression values obtained using the PATseq dataset. 1 to 9 represent sample IDs.

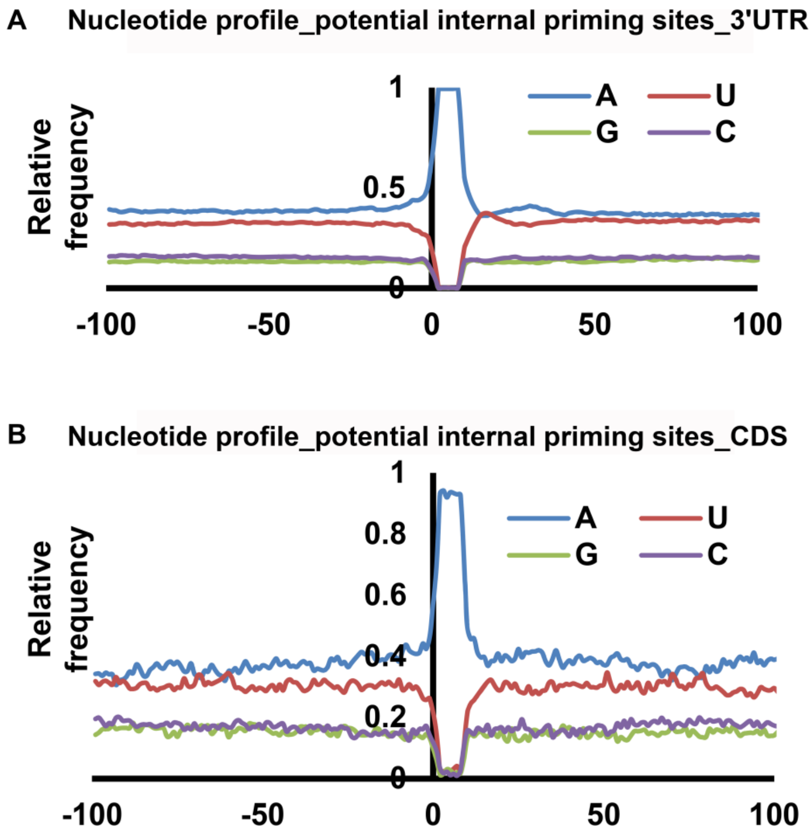

Supplemental Fig.S3. Nucleotide composition of the sequences surrounding potential internal priming sites. Potential internal priming sites are defined by 'N6A8' motif, where 'N' is any nucleotide, followed by a stretch of eight or more consecutive 'A's. Sequences 100 nucleotides upstream and downstream of potential internal priming sites were used for the analysis. **A and B** represent single nucleotide profiles of the sequences surrounding potential internal priming sites located at 3'UTR and coding regions, respectively in the red clover genome. Nucleotide profiles were analyzed using the pooled samples of all three tissue types. Y-axis represents relative frequency of each nucleotide.

Supplemental Table.S1. Mapping statistics of PATseq libraries

Sample ID	Tissue type	Barcode	Mapped reads
1	Leaf	CCCTTTT	1,561,379
2	Root	AACTTTT	544,778
3	Flower	AGCTTTT	892,817
4	Leaf	ACGTTTT	3,032,588
5	Root	AGATTTT	1,136,652
6	Flower	CCGTTTT	1,846,965
7	Leaf	CAATTTT	4,007,793
8	Root	CAGTTTT	1,418,290
9	Flower	GGATTTT	3,013,933
**Total mapped reads**			**17,455,195**
**Average mapped reads**			**1,939,466**

Supplemental Table.S2. Statistics of PATs extracted from the RNAseq libraries

RNAseq library	Tissue type	RNAseq reads	Non-poly(A) reads	Poly(A)-tail containing reads	Trimmed PATs
Tp_RNAseq_1	Leaf	14276353	14275230	1123	1067
Tp_RNAseq_2	Root	15457898	15457017	881	846
Tp_RNAseq_3	Flower	8273183	8272747	436	414
Tp_RNAseq_4	Leaf	5522441	5521661	780	716
Tp_RNAseq_5	Root	15091453	15090286	1167	1094
Tp_RNAseq_6	Flower	17331617	17331369	248	240
Tp_RNAseq_7	Leaf	7466841	7464841	2000	1930
Tp_RNAseq_8	Root	9153237	9152562	675	625
Tp_RNAseq_9	Flower	14862895	14862581	314	286
**Total**		**107435918**	**107428294**	**7624**	**7218**

Supplemental Table.S3. Pearson correlation coefficients between replicates of PATseq libraries

Comparison	Pearson correlation coefficient
L1 vs L4	0.86
L1 vs L7	0.87
L4 vs L7	0.92
R2 vs R5	0.89
R2 vs R8	0.85
R5 vs R8	0.88
F3 vs F6	0.86
F3 vs F9	0.85
F6 vs F9	0.86
